# Aspirin-triggered 15-epi-lipoxin A_4_ predicts cyclooxygenase-2 in the lungs of LPS-treated mice but not in the circulation: implications for a clinical test

**DOI:** 10.1096/fj.12-215533

**Published:** 2013-10

**Authors:** Nicholas S. Kirkby, Melissa V. Chan, Martina H. Lundberg, Karen A. Massey, William M. B. Edmands, Louise S. MacKenzie, Elaine Holmes, Anna Nicolaou, Timothy D. Warner, Jane A. Mitchell

**Affiliations:** * National Heart and Lung Institute, Imperial College London, London, UK;; † William Harvey Research Institute, Barts and the London School of Medicine, London, UK;; ‡ School of Pharmacy, University of Bradford, Bradford, UK, and; § School of Life and Medical Sciences, University of Hertfordshire, Hatfield, UK

**Keywords:** 15-HETE, COX-2 biomarker, vascular inflammation, nonsteroidal anti-inflammatory drugs

## Abstract

Inhibition of cyclooxygenase (COX)-2 increases cardiovascular deaths. Identifying a biomarker of COX-2 is desirable but difficult, since COX-1 and COX-2 ordinarily catalyze formation of an identical product, prostaglandin H_2_. When acetylated by aspirin, however, COX-2 (but not COX-1) can form 15(*R*)-HETE, which is metabolized to aspirin-triggered lipoxin (ATL), 15-epi-lipoxin A_4_. Here we have used COX-1- and COX-2-knockout mice to establish whether plasma ATL could be used as a biomarker of vascular COX-2 *in vivo*. Vascular COX-2 was low but increased by LPS (10 mg/kg; i.p). Aspirin (10 mg/kg; i.v.) inhibited COX-1, measured as blood thromboxane and COX-2, measured as lung PGE_2_. Aspirin also increased the levels of ATL in the lungs of LPS-treated wild-type C57Bl6 mice (vehicle: 25.5±9.3 ng/ml; 100 mg/kg: 112.0±7.4 ng/ml; *P*<0.05). Despite this, ATL was unchanged in plasma after LPS and aspirin. This was true in wild-type as well as *COX-1*^−/−^ and *COX-2*^−/−^ mice. Thus, in mice in which COX-2 has been induced by LPS treatment, aspirin triggers detectable 15-epi-lipoxin A_4_ in lung tissue, but not in plasma. This important study is the first to demonstrate that while ATL can be measured in tissue, plasma ATL is not a biomarker of vascular COX-2 expression.—Kirkby, N. S., Chan, M. V., Lundberg, M. H., Massey, K. A., Edmands, W. M. B., MacKenzie, L. S., Holmes, E., Nicolaou, A., Warner, T. D., Mitchell, J. A. Aspirin-triggered 15-epi-lipoxin A_4_ predicts cyclooxygenase-2 in the lungs of LPS-treated mice but not in the circulation: implications for a clinical test.

Cyclooxygenase (COX) enzymes catalyze the conversion of arachidonic acid to prostaglandin H_2_, an unstable intermediate, which is the precursor to a range of end-product prostanoid mediators, including prostacyclin (PGI_2_), thromboxane A_2_ (TXA_2_), and prostaglandin (PG) E_2_. Two isoforms of COX exist (1). COX-1 is constitutively expressed in most tissues and produces prostanoids responsible for normal physiological functions such as hemostasis and mucosal integrity. COX-2 is primarily considered to be an inducible isoform. It is sparsely present in most healthy tissues (1), but is up-regulated by inflammatory and mitogenic stimuli and produces prostanoids that potentiate pain and inflammation. Inhibitors of COX-2, both traditional nonsteroidal anti-inflammatory drugs (NSAIDs), such as ibuprofen and diclofenac, which inhibit both COX-1 and COX-2, and newer selective COX-2 agents, such as rofecoxib and celecoxib (2), have been widely used for the treatment of arthritis and other inflammatory conditions. Although they are effective for these indications, it has become clear that chronic use of inhibitors of COX-2 (both selective and traditional NSAIDs) is associated with an increased risk of cardiovascular events, particularly myocardial infarction (3). The mechanisms responsible for this cardiovascular toxicity are not fully understood but may reflect loss of cardioprotective prostanoid pathways in particular patients in whom COX-2 is abnormally present in the vascular wall of the renal, cardiac, or systemic circulation. This raises the important possibility that screening for cardiovascular COX-2 expression may identify specific individuals at higher or lower risk of NSAID-induced cardiovascular toxicity.

The accepted standard for assessing prostanoid production *in vivo* is the measurement of urinary prostanoid metabolites. The true source of these metabolites is not clear, however, and it is being increasingly questioned whether these markers reflect systemic prostanoid biosynthesis (4–6) or a more localized pool. Indeed, we have recently shown that while COX-2 drives urinary markers of PGI_2_, COX-1 is responsible for levels in the circulation (5). Most importantly, measurements of regular COX metabolites can never irrefutably distinguish COX-1 from COX-2-dependent synthetic pathways because both isoforms normally catalyze an identical reaction. Aspirin inhibits the ability of both isoforms to convert arachidonic acid to PGH_2_ by acetylation of a key serine residue in the active site (7). In the presence of aspirin, however, the catalytic activity of COX-1 and COX-2 is differently altered (8, 9). Specifically, *in vitro* aspirin-acetylated COX-2 gains the ability to convert arachidonic acid to 15(*R*)-hydroxyeicosatetraenoic acid [15(*R*)-HETE; refs. 8–10]. Further, in endothelial cell and leukocyte cocultures, endothelial cell-derived 15(*R*)-HETE can undergo transcellular metabolism by leukocyte 5-lipoxygenase (5-LO) to form 15-epi-lipoxin A_4_ (10), a novel lipid mediator reported to possess proresolution activity in situations of inflammation (11). As far as is known, this pathway is absolutely dependent on aspirin-acetylated COX-2 and cannot be mediated by COX-1. In addition, COX-independent pathways for 15(*R*)-HETE and 15-epi-lipoxin A_4_ formation have been described involving cytochrome P450 enzymes (12), nonenzymatic oxidation of arachidonic acid (13), or phosphorylated 5-LO (14). These may be enhanced by statins (14, 15) but are not triggered by aspirin. Here, we have investigated whether aspirin-triggered formation of 15-epi-lipoxin A_4_ [aspirin-triggered lipoxin (ATL)] could provide a plasma biomarker of vascular COX-2 activity. We reveal that while ATL can be detected in mouse plasma, levels do not correlate with the expression level of COX-2 in the vasculature, suggesting that ATL is not a suitable means for the noninvasive screening of patients for vascular COX-2 expression.

## MATERIALS AND METHODS

### Animals

*COX-1*^−/−^ (16) and *COX-2*^−/−^ mice (17) were backcrossed for >7 generations onto a C57Bl/6 background (Harlan Bioproducts, Bicester, Oxon, UK). Wild-type (WT) mice were generated by intercrossing C57Bl/6 backcrossed *COX-1*^+/−^ and *COX-2*^+/−^ mice. All mice used in the study were genotyped to establish COX-1 and COX-2 status before use. All experiments were performed on male and female 10- to 12-wk old mice and were conducted in accordance with Animals (Scientific Procedures) Act 1986 and after local ethical review by the Imperial College London Ethical Review Panel (PPL 70/10546).

### Whole blood, lung, and aorta COX activity assays

WT, *COX-1*^−/−^, and/or *COX-2*^−/−^ mice were euthanized with CO_2_. In some cases, 30 min beforehand, animals were treated with aspirin-lysine (Flectadol; Sanofi-Aventis, Milan, Italy) or vehicle by tail-vein injection. Lungs were removed and snap-frozen. Blood was collected from the inferior vena cava into lithium heparin (17 U/ml final; Sigma-Aldrich, Gillingham, UK), then stimulated with calcium ionophore A23187 (50 μM; Sigma-Aldrich) for 30 min at 37°C. Plasma was separated by centrifugation and the levels of the stable TXA_2_ breakdown product, TXB_2_, determined by enzyme immunoassay (Cayman Chemical, Ann Arbor, MI, USA).

Thoracic aorta was removed and carefully cleaned of periadventitial material. Next, 2 mm rings of aorta were divided and allowed to equilibrate in DMEM (Sigma-Aldrich) for 90 min, before stimulation with calcium ionophore A23187 (50 μM). After 30 min, conditioned medium was removed for measurement of the stable prostacyclin breakdown product 6-keto-PGF_1α_ by ELISA (Cayman Chemical).

Lungs were dissociated using a ceramic bead homogenizer (Precellys 24; Bertin Technologies, Rockville, MD, USA) in Tris buffer (50 mM; Sigma-Aldrich) containing phenylmethanesulfonyl fluoride (1 mM; Sigma-Aldrich) and diclofenac (1 mM; Sigma-Aldrich). Supernatants were separated by centrifugation and collected for measurement of PGE_2_ using a homogenous time-resolved fluorescence-based immunoassay (Cisbio Bioassays, Codolet, France) and for 6-keto-PGF_1α_ using ELISA as described above.

### Circulating prostacyclin measurement

WT, *COX-1*^−/−^, and/or *COX-2*^−/−^ mice were treated with lipopolysaccharide (LPS; 10 mg/kg i.p.; from Escherichia coli 055:B5; Sigma-Aldrich) or vehicle (saline). After 4 h, mice were euthanized with CO_2_, and blood was collected from the inferior vena cava. Plasma was separated by centrifugation, and the levels of the PGI_2_ breakdown product, 6-keto-PGF_1α_, were determined by ELISA (Cayman Chemical).

### COX immunohistochemistry

WT mice treated with LPS (10 mg/kg) or vehicle by intraperitoneal injection were euthanized with CO_2_ after 4 h, and the vasculature was fixed with 5% neutral buffered formalin (Sigma-Aldrich) by transcardiac perfusion. The thoracic aorta was carefully removed for whole mount immunohistochemistry. Briefly, vessels were treated with 20% goat serum and 0.1% Triton X-100 to block nonspecific binding and permeabilize cells, respectively. They were then incubated, in turn, with rabbit anti-mouse COX-2 primary antibody (1:50; 4 h; Cayman Chemical), Alexa594-conjugated goat anti-mouse IgG secondary antibody (1:100; 2 h; Invitrogen, Paisley, UK), and Alexa488-conjugated rat anti-mouse CD31 antibody (1:100; 2 h; BioLegend, London, UK) with thorough washing with PBS between reagents. Vessels were cut open and mounted flat using hardset aqueous medium (Vector Labs, Peterborough, UK). The luminal surface was visualized using a Leica SP5 inverted confocal microscope and a ×40 oil-immersion objective lens (Leica Microsystems, Wetzlar, Germany). The endothelial cell layer was identified based on cell morphology and CD31 immunoreactivity, and the background corrected fluorescence intensity of COX-2-like immunoreactivity was quantified using Fluorescence Lite software (Leica). The specificity of the staining was confirmed by the ability of COX-2 gene deletion or specific blocking peptides to quench immunoreactivity (5).

### qRT-PCR

WT mice treated with LPS (10 mg/kg) or vehicle by intraperitoneal injection were euthanized with CO_2_ after 0, 4, 12, 18, or 24, h and the lung and aorta were immediately removed and snap-frozen. Tissue was homogenized as above, and RNA was extracted using a silica column-based kit (Invitrogen). cDNA was synthesized by reverse transcription (SuperScript II; Invitrogen) with oligo-d(T) primers. cDNA was amplified and quantified by qPCR using TaqMan gene expression assays (18S: Mm03928990_g1; PTGS2: Mm00478374_m1; Invitrogen) and a Rotor Gene Q instrument (Qiagen Ltd., Manchester, UK). COX-2 (Ptgs2) expression levels were normalized to 18S ribosomal RNA levels and quantified using the comparative *C*_*t*_ method.

### Myeloperoxidase measurement

WT mice treated with LPS (10 mg/kg) or vehicle by intraperitoneal injection were euthanized with CO_2_ after 0, 4, 12, 18 or 24 h, and the lung was immediately removed and snap-frozen. Tissue was homogenized in PBS as above, and the myeloperoxidase content of the supernatant determined by enzyme immunoassay (R&D Systems, Minneapolis, MN, USA).

### Lipoxin A_4_ and ATL measurement by ELISA

WT, *COX-1*^−/−^ and *COX-2*^−/−^ mice (each *n*=8) were treated with LPS (10 mg/kg; i.p.) or vehicle. After 4 h, mice additionally received either aspirin-lysine (10 or 100 mg/kg; i.v.) or its vehicle. For time-course studies, WT mice (*n*=4) were treated with LPS (10 mg/kg; i.p.) or vehicle for 0, 4, 12, 18, or 24 h before administration of aspirin-lysine (100 mg/kg; i.v.) or its vehicle. After a further 30 min, mice were euthanized with CO_2_. Venous blood was collected into lithium heparin, and plasma was separated by centrifugation. Lungs and aorta were also collected and snap-frozen before disruption with a Precellys24 homogenizer (100 mg tissue/ml) in Tris buffer (50 mM; pH 7.4) with phenylmethanesulfonyl fluoride (1 mM). Levels of lipoxin A_4_ and 15-epi-lipoxin A_4_ in plasma and the supernatant of lung and aorta homogenates were determined by enzyme immunoassay (Neogen Corp., Lansing, MI, USA). To exclude any matrix interference effects, samples were assayed for 15-epi-lipoxin A_4_ both after HPLC extraction using C18 columns, as recommended by the assay manufacturer, and after dilution 1:9 with assay buffer. For HPLC/C18 extraction, 50–100 μl of plasma was diluted in methanol-water (15%) to a final volume of 1 ml and acidified to pH 3.5. Samples were centrifuged (5°C 4000 *g*) and supernatant applied to a Waters HPLC SunFire reversed phase Silica C_18_ column (Waters Corp., Wexford, Ireland). Following washing with water and hexane, samples were eluted using methyl formate, and samples were centrifuged before supernatants were evaporated under a stream of nitrogen. Samples were reconstructed with ELISA buffer as instructed. In addition, a subset of plasma samples (*n*=4) was subjected to chiral analysis by liquid chromatography-tandem mass spectrometry (chiral lipid analysis; LC-MS/MS) to determine the levels of 12(*S*)-HETE, 12(*R*)-HETE, 15(*S*)-HETE, 15(*R*)-HETE, LXA_4_, and 15-epi-lipoxin A_4_ according to the method described below (18).

### Chiral chromatography/mass spectrometry

Levels of 12(*S*)-HETE, 12-(*R*)-HETE, 15-(*S*)-HETE, 15-(*R*)-HETE, LXA_4_, and 15-epi-lipoxin A_4_ in plasma from mice treated with the above combinations of LPS and aspirin-lysine were analyzed using chiral liquid chromatography coupled to electrospray ionization tandem mass spectrometry (LC/ESI-MS/MS) as described previously (18). Briefly, plasma samples 200–450 μl) were defrosted on ice and adjusted to 15% v/v methanol, to a final volume of 4 ml. Internal standard 12(*S*)-HETE-*d*8 (40 ng; Cayman Chemical) was added. Samples were acidified to pH 3.0 using 0.1 M HCl and then directly loaded onto a preconditioned solid-phase extraction cartridge (C18-E; Phenomenex, Cheshire, UK). Lipid mediators were eluted with methyl formate. Chiral chromatography was performed on a LUX-1 cellulose column (3 μm, 150×2.0 mm; Phenomenex) using an HPLC pump (Alliance 2695; Waters) coupled to a triple-quadrupole mass spectrometer with electrospray probe (Quatro Ultima; Waters). Lipid mediators were analyzed in multiple reaction monitoring (MRM) mode using the following transitions: 15(*R*)-HETE and 15(*S*)-HETE *m/z* 319 > 175, 12(*R*)-HETE and 12(*S*)-HETE *m/z* 319 > 179, LXA_4_ and 15-epi LXA_4_ *m/z* 351 > 115, and 12(*S*)-HETE-*d*8 *m/z* 327 > 184. Results are expressed as picograms of mediator per microliter of plasma.

### Statistics and data analysis

Data were analyzed using Prism 5.01 for Mac OS X (GraphPad Software, San Diego, CA, USA). Statistical significance was determined by 1-way ANOVA with Dunnett's *post hoc* test unless otherwise stated, and data sets were considered different if *P* < 0.05.

## RESULTS AND DISCUSSION

### COX-1 *vs.* COX-2 activity in control and LPS-treated mice

**Figure 1.**
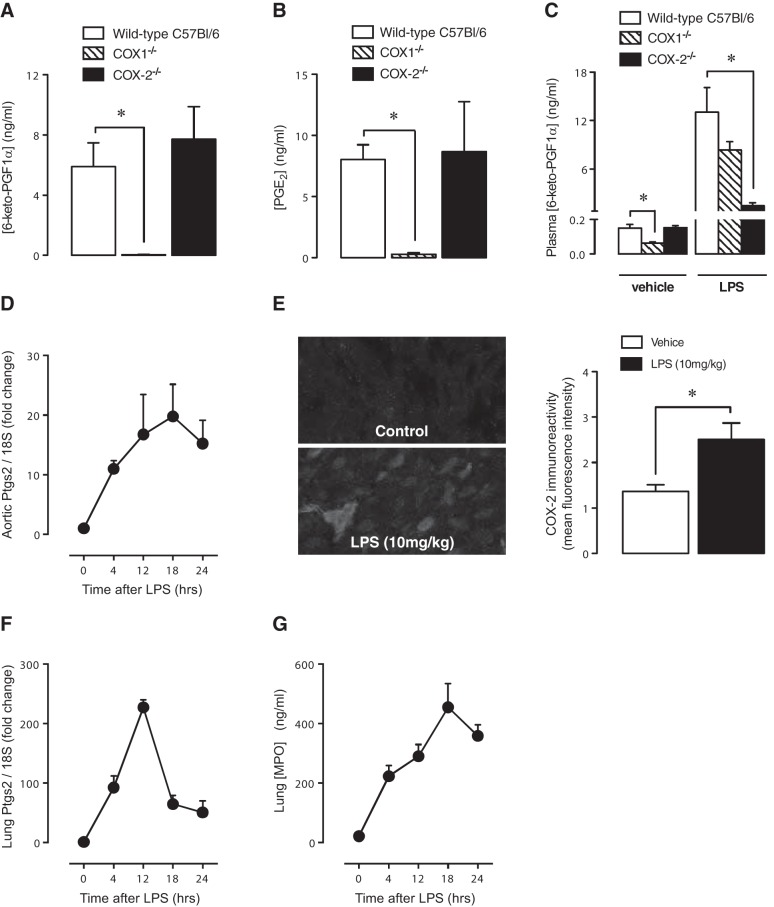
COX-2 expression activity in the vasculature and the effect of LPS. In untreated mice, Ca^2+^ ionophore-stimulated prostacyclin release (measured as 6-keto-PGF_1α_) by aortic segments (*A*) and PGE_2_ formation in lung homogenates (*B*) was not altered by COX-2 deletion, but abolished by COX-1 deletion. Accordingly, basal plasma levels of 6-keto-PGF_1α_ were reduced by COX-1 but not COX-2 deletion (*C*). Administration of LPS (10 mg/kg) increased plasma prostacyclin levels (*C*), and this effect was COX-2 dependent. LPS also produced a time-dependent increased in aortic COX-2 (Ptgs2) gene expression (*D*), and this was detectable at 4 h as an increased COX-2-like immunoreactivity (*E*). LPS also produced a time-dependent increased in lung COX-2 gene expression (*F*) and neutrophil infiltration into the lung, measured as increased myeloperoxidase levels (*G*). Data are the means ± se for tissue from *n* = 4–8 mice aged 10–12 wk. Data were analyzed using unpaired *t* test (*E*) or 1-way ANOVA followed by Bonferroni's multiple comparison test (*A–C*). *P < 0.05.

The generation of 15(*R*)-HETE or downstream ATL by aspirin *in vitro* relies on the presence of COX-2. However, we have recently shown in human endothelium and in vessels of healthy mice that COX-1 rather than COX-2 predominates (5). In the current study, we confirm our previous observations that COX activity in the vessel (**Fig. 1*A***) or in the lung (Fig. 1*B*) is overwhelmingly COX-1 driven (5). Similarly, as we (5) and others (19) have shown, basal release of PGI_2_ in control mice measured in plasma *ex vivo* is driven by COX-1 (Fig. 1*C*). To test our hypothesis that ATL could be a viable biomarker of circulatory COX-2, we therefore treated mice with LPS to increase COX-2 expression in the endothelium above low-level basal expression. LPS at 10 mg/kg produced a time-dependent increased in aortic COX-2 (*Ptgs2*) gene expression, between 4 and 24 h (Fig. 1*D*), and this was detectable as an increase in COX-2-like immunoreactivity in the aorta by 4 h (Fig. 1*E*). In parallel, at 4 h after LPS, plasma prostacyclin was increased by ≈60 fold, which, in direct contrast to prostacyclin under basal conditions, was predominately COX-2 driven (Fig. 1*C*). Similar to the aorta, lung COX-2 gene expression was increased by LPS between 4 and 24 h, peaking at 12 h (Fig. 1*F*), and this was accompanied by an increase in leukocyte infiltration into the lung, measured as myeloperoxidase content (Fig. 1*G*).

### Plasma levels of ATL and HETEs in control mice and LPS treated mice

**Figure 2.**
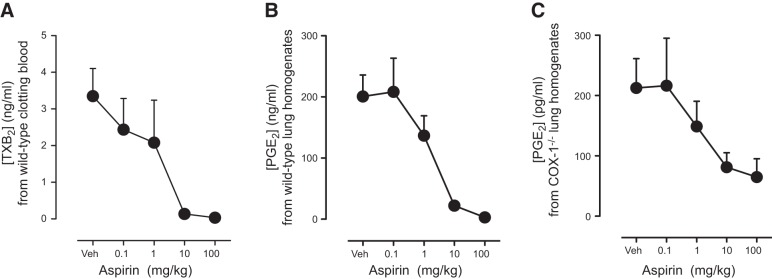
Inhibition of COX-1 and COX-2-dependent prostanoid formation by aspirin *in vivo*. Aspirin administration (0.1–100 mg/kg, i.v.; 30 min) produced dose-dependent inhibition of serum TXB_2_ formation (*A*) and lung homogenate PGE_2_ formation (*B*) in tissue from WT mice, both responses that are primarily COX-1 dependent. Aspirin also produced dose-dependent inhibition of PGE_2_ formation in lung homogenates from COX-1-deficient mice, in which residual prostanoid production (∼0.1% of WT levels) is mediated by COX-2 (*C*). In all systems, aspirin produced >70% inhibition of COX activity at 100mg/kg. Data are means ± se for tissue from *n* = 3–5 mice aged 10–12 wk.

In rats (20) or mice (21), aspirin administration increases ATL. Moreover, in healthy volunteers taking low-dose but not medium- or high-dose aspirin for 8 wk, increases in ATL in plasma were noted (22). These observations provided the impetus for our study. However, to demonstrate the suitability of ATL as a useful biomarker of COX-2 in the circulation in patients with active cardiovascular and/or inflammatory disease, we required a system wherein ATL could be measured acutely after aspirin and levels detectably increased with COX-2 induction. We first wanted to establish a pharmacological dose of aspirin that would effectively inhibit COX-1 and COX-2 in mice *in vivo*. This was particularly important to do since higher doses of aspirin were found to antagonize ATL generation in healthy human volunteers (22) and because mice are relatively insensitive to the effects of aspirin (21, 23). The potency of aspirin administered i.v. on COX-1 activity *in vivo* was measured by thromboxane formation in clotting blood (**Fig. 2*A***) and PGE_2_ in lung homogenates *ex vivo* (Fig. 2*B*). The potency of aspirin on COX-2 activity *in vivo* was measured as PGE_2_ in lung homogenate of COX-1^−/−^ mice (Fig. 2*C*). Effective inhibition of COX-1 (Fig. 2*A*, *B*) or COX-2 (Fig. 2*C*) was seen at aspirin concentrations of 10 mg/kg and greater.

**Figure 3.**
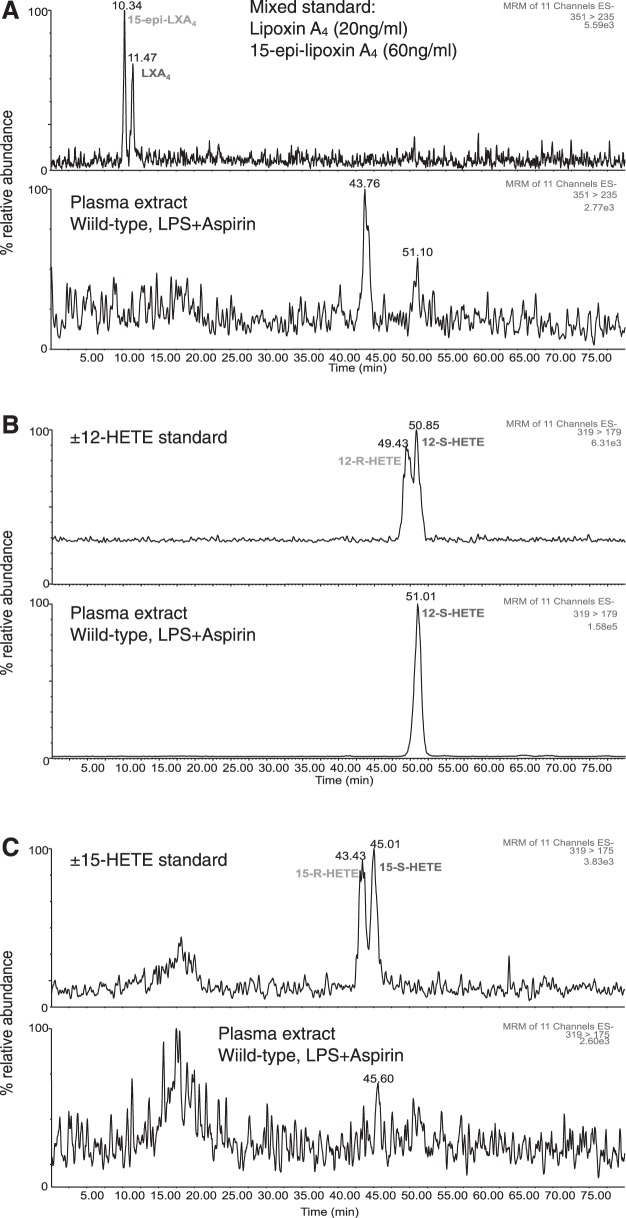
Aspirin treatment in LPS-induced mice does not produce detectable ATL formation in WT mouse plasma. Representative chromatograms for the chiral analysis of the ATL, 15-epi-lipoxin A_4_, lipoxin A_4_ (*A*) 12(*R/S*)-HETE, (*B*) and 15(*R/S*)-HETE (*C*) by LC-MS/MS. Endogenous ATL could not be detected in plasma from WT mice treated with LPS and aspirin (10 mg/kg) by chiral LC-MS/MS. Only 12(*S*)-HETE, not 12(*R*)-HETE or 15(*R/S*)-HETE, was detectable in plasma from LPS-treated mice. Chromatograms are representative of *n* = 4 independent experiments.

In control mice, despite effectively blocking COX activity, aspirin (10 mg/kg) did not increase plasma levels of ATL, as measured by ELISA (**Tables 1** and **2**). As discussed above, control mice express very low levels of COX-2 activity, but this could be increased by treatment with LPS (Fig. 1). Even in mice treated with LPS for 4 to 24 h to induce COX-2, however, aspirin (10 mg/kg) did not increase ATL in plasma (Table 1). Similar results were obtained measuring ATL in aortic homogenates (Table 1). Further, COX-1 or -2 deletion did not alter plasma ATL levels (Table 2), suggesting either that basal levels are derived from a COX-independent pathway (15) or are an artifact of ELISA-based measurement. Our analysis of ATL using ELISA was further explored using chiral lipid analysis by LC-MS/MS (**Fig. 3**; Table 2). Analysis of ATL and LXA_4_ standards showed elution peaks at 10.34 and 11.47 min, respectively (Fig. 3*A*). No corresponding peaks were seen in the plasma of LPS- and aspirin-treated WT mice (Fig. 3 and Table 2). Our detection of ATL by ELISA but not chiral LC-MS/MS may be explained by the levels of sensitivity of these two assays as used: detection limits were 0.2 ng/ml of the ELISA and 2 ng/ml for chiral analysis (18).

**Table 1.** Aspirin does not alter plasma of aortic levels of ATL (15-epi-lipoxin A_4_) or LXA_4_ (measured by ELISA) between 0 and 24 h after LPS treatment

Treatment	LXA_4_ control (ng/ml)	LXA_4_ aspirin (ng/ml)	ATL control (ng/ml)	ATL aspirin (ng/ml)
Plasma				
Control, 0 h	1.6 ± 0.3	1.5 ± 0.3	6.0 ± 0.7	4.8 ± 0.5
LPS				
4 h	6.1 ± 0.8^*^	5.1 ± 0.8^*^	7.4 ± 1.1	7.3 ± 1.3
12 h	4.3 ± 0.7	5.7 ± 0.8^*^	6.4 ± 0.8	7.0 ± 1.8
18 h	4.0 ± 0.4	5.7 ± 1.0^*^	5.1 ± 0.5	7.5 ± 1.6
24 h	2.6 ± 0.2	4.6 ± 0.7	3.8 ± 0.8	4.6 ± 0.4
Aorta homogenate				
Control, 0 h	8.9 ± 1.3	4.5 ± 0.7	23.4 ± 4.6	17.9 ± 5.6
LPS				
4 h	7.6 ± 0.7	7.8 ± 1.7	28.7 ± 1.9	31.1 ± 8.4
12 h	6.4 ± 1.1	8.7 ± 1.4	29.7 ± 7.6	41.5 ± 9.2
18 h	5.4 ± 1.1	5.4 ± 0.5	18.6 ± 4.3	20.2 ± 2.8
24 h	3.9 ± 0.5	6.6 ± 0.4	12.8 ± 2.3	24.8 ± 0.6

Plasma or aortic ATL levels were not altered by aspirin (100 mg/kg; 30 min) when measured by commercial ELISA between 0 and 24 h after LPS treatment. Plasma but not aorta LXA_4_ levels were increased by LPS but not altered by aspirin. Data are the means ± se for tissue from *n* = 4 mice aged 10–12 wk. Data were analyzed for the effect of time point and aspirin using 2-way ANOVA followed by Tukey's multiple comparison test.

* *P* < 0.05 *vs.* control.

**Table 2.** COX-1 or COX-2 deletion does not alter plasma levels of ATL (15-epi-lipoxin A_4_, measured by ELISA) in control or LPS-primed mice treated with aspirin

Genotype	ATL (ng/ml)			
	Vehicle	Aspirin	LPS	LPS + aspirin
Diluted plasma				
WT C57Bl/6	1.2 ± 0.1	1 ± 0.2	1.6 ± 0.2	1.2 ± 0.1
*COX-1*^−/−^	1.5 ± 0.1	1.3 ± 0.1	1.4 ± 0.1	1.2 ± 0.1
*COX-2*^−/−^	1.5 ± 0.3	1.3 ± 0.2	1.6 ± 0.1	1.4 ± 0.1
HPLC-extracted plasma				
WT C57Bl/6	1.9 ± 0.2	1.8 ± 0.2	2.3 ± 0.3	1.9 ± 0.3
*COX-1*^−/−^	1.7 ± 0.2	1.8 ± 0.1	2.0 ± 0.2	1.8 ± 0.2
*COX-2*^−/−^	2.1 ± 0.2	2.1 ± 0.2	2.1 ± 0.4	2.0 ± 0.3

Plasma ATL levels were not altered by aspirin (10 mg/kg), LPS, or COX-1/2 deletion when measured by commercial ELISA in plasma diluted in buffer or extracted using HPLC and C18 columns. Data are the means ± se for tissue from *n* = 8 mice aged 10–12 wk. Data were analyzed within each genotype using 1-way ANOVA followed by Bonferroni's multiple comparison test.

In addition to COX-2 acetylation, the generation of COX-derived ATLs requires activation of phospholipase A_2_ to liberate the substrate arachidonic acid, and presence of neutrophil 5-LO to metabolize 15(*R*)-HETE to ATL. Clearly, in our mice treated with LPS, we have elevated (compared to control mice) levels of COX-2 (Fig. 1). Both 12(*S*)-HETE and lipoxin A_4_ are metabolites of arachidonic acid, and, as such, their formation requires activation of phospholipase A_2_. In the absence of ATL being detected, increases in 12(*S*)-HETE (**Table 3**) and lipoxin A_4_ (Table 1) in the plasma of LPS-treated mice therefore provide important evidence that phospholipase A_2_ is activated in our animals *in vivo*. Our inability to detect LPS-induced changes in plasma lipoxin A_4_ by chiral LC-MS/MS is likely to reflect the lower sensitivity of this method (18).

**Table 3.** Quantitation of plasma ATL (15-epi-lipoxin A_4_), LXA_4_, 12(*R/S*)-HETE, and 15(*R/S*)-HETE levels by chiral chromatography mass spectrometry in plasma from aspirin (10 mg/kg)- and LPS (10 mg/kg)-treated mice

Component	Vehicle	Aspirin	LPS	LPS + aspirin
ATL (ng/ml)	ND	ND	ND	ND
LXA_4_ (ng/ml)	ND	ND	ND	ND
12(*S*)-HETE (ng/ml)				
WT C57Bl/6	13.0 ± 2.2	12.0 ± 2.7	36.0 ± 7.3	38.2 ± 10.5^*^
*COX-1*^−/−^	11.5 ± 1.3	10.7 ± 1.9	37.6 ± 5.8^*^	35.4 ± 11.5
*COX-2*^−/−^	12.3 ± 3	11.1 ± 2.8	27.8 ± 13.7	36.8 ± 7.5
12(*R*)-HETE (ng/ml)	ND	ND	ND	ND
15(*S*)-HETE (ng/ml)	ND	ND	ND	ND
15(*R*)-HETE (ng/ml)	ND	ND	ND	ND

ATL, LXA_4_, 12(*R*)-HETE, 15(*S*)-HETE, and 15(*R*)-HETE were not detectable (ND) in plasma from any tested treatment group and genotype of mice. 12(*S*)-HETE was detectable in all plasma samples and tended to be increased by LPS administration in all genotypes of mice, consistent with the ability of LPS to increase phospholipase A_2_ activity *in vivo*. Data are the means ± se for tissue from *n* = 4 mice aged 10–12 wk. Data were analyzed within each genotype using 1-way ANOVA followed by Bonferroni's multiple comparison test.

* *P* < 0.05 *vs*. vehicle.

Again, in the absence of ATL, we wanted to determine whether the upstream aspirin triggered metabolite, 15(*R*)-HETE, could be used as a plasma biomarker for increased COX-2 in the vasculature. We eluted 15(*R*)-HETE and 15(*S*)-HETE at 43.43 and 45.01 min, respectively, but neither metabolite was detected in mouse plasma (Fig. 3*C*), suggesting that they were at or below the limit of detection for the assay (2 ng/ml; Table 3).

### ATL in lungs of control and LPS treated mice; comparison with plasma levels

**Figure 4.**
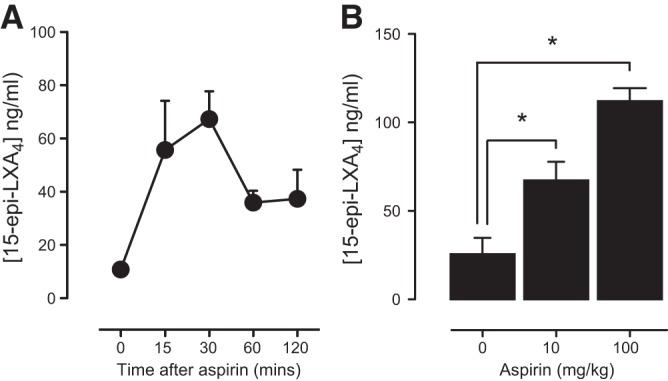
Aspirin produces dose- and time-dependent increases in ATL (15-epi-lipoxin A_4_) levels in the lung of WT mice. Administration of aspirin (10 mg/kg) to LPS-primed WT mice produced an increase in ATL levels in lung homogenates (*A*), which peaked at 30 min then declined slowly. In independent experiments, in LPS-primed mice, lung ATL levels 30 min after aspirin administration were found to increase in a dose-dependent manner (*B*). Data are the means ± se for tissue from *n* = 4–8 mice aged 10–12 wk. Data were analyzed using 1-way ANOVA followed by Bonferroni's multiple comparison test. *P < 0.05.

**Figure 5.**
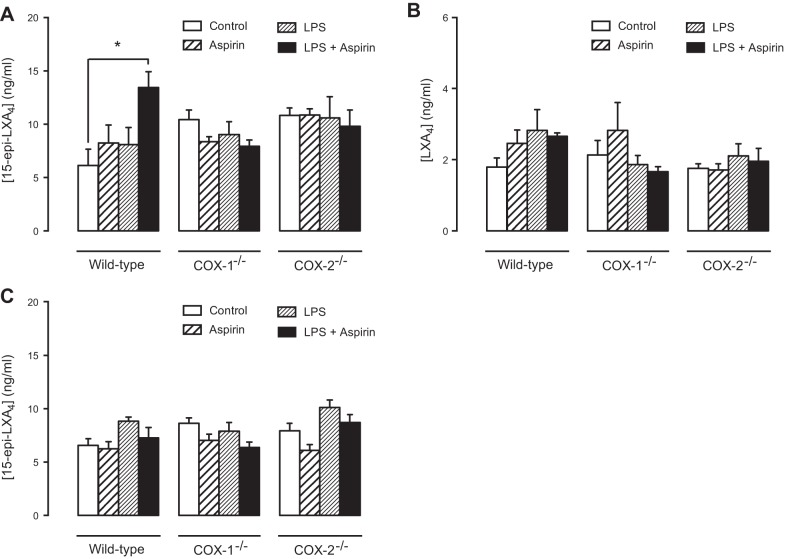
ATL (15-epi-lipoxin A_4_) formation in lung homogenates is COX-2 dependent. In lung homogenates from WT mice, administration of aspirin (100 mg/kg) and LPS (10 mg/kg) but neither on its own, produced an increase in ATL levels (*A*) but not LXA_4_ levels (*B*). This effect was abolished by deletion of COX-2 or COX-1. In the same animals, ATL levels in plasma were not altered by any treatment or COX-1/2 deletion (*C*). Data are the means ± se for tissue from *n* = 6 mice aged 10–12 wk. Data were analyzed using 1-way ANOVA followed by Bonferroni's multiple comparison test. *P < 0.05.

Aspirin-triggered 15(*R*)-HETE is generated by microsomal fractions of cells expressing COX-2 (24) and by COX-2-expressing human endothelial cells (10) or lung epithelial cells (25). ATL is generated when 15(*R*)-HETE is further metabolized by 5-LO (25). This can be modeled *in vitro* by coculture of endothelial or epithelial cells with monocytes or neutrophils (10, 20, 25, 26). While basal levels of ATL were detected by ELISA in the plasma of mice used in our study, we were not able to demonstrate significant increases in animals where COX-2 had deliberately been increased using LPS. To provide a positive control for our studies and to better understand how this reflects generation of ATLs in an optimized system, we measured ATL formation in the lung of LPS-treated mice. Here an optimal setting exists where endothelial cell/epithelial cells in which COX-2 is induced by LPS (Fig. 1*D*) are in contact with primed leukocytes, whose presence was confirmed by a ∼10-fold increase in lung myeloperoxidase content (Fig. 1*G*). Consistent with what has been previously shown in inflamed mouse tissue (26), we found increased ATL levels in the lungs of mice treated with aspirin (100 mg/kg) after LPS (**Fig. 4**) in parallel with increased COX-2 gene expression (Fig. 1). Notably, ATL levels were increased in a dose-dependent manner (100>10 mg/kg; Fig. 4*B*) and release (aspirin: 10 mg/kg) was transient, with peak levels being detected at 30 min after aspirin administration (Fig. 4*A*). In a separate study using the high dose of 100 mg/kg of aspirin, designed to directly parallel measurement of ATL in the lungs and plasma of WT and COX-1/2-knockout mice, we again found that aspirin increased ATL (**Fig. 5*A***) but not LXA_4_ in the lungs (Fig. 5*B*), or ATL in plasma of LPS-primed WT mice (Fig. 5*C*). This effect was lost in tissue from *COX-2*^−/−^ mice, illustrating that ATL generation was dependent on COX-2 (Fig. 5*A*) rather than non-COX pathways, such as cytochrome P450s, consistent with previous studies in inflamed mouse tissue (26). However, we also found that the ability of aspirin to increase ATL was also lost in tissue from *COX-1*^−/−^ mice. The explanation for this is not clear but may reflect a role for COX-1 in mediating early inflammatory events (16) that may be required to assemble relevant cell types in proximity (*e.g.*, endothelial cells and leukocytes).

## CONCLUSIONS

These studies are the first to directly address the potential for plasma levels or ATL as a biomarker for COX-2 in the circulation. In control mice, COX-1 drives circulating (plasma) PGI_2_ levels. However, when mice are treated with LPS, COX-2 is induced and takes a major role in circulating levels of PGI_2_. Basal levels of ATL are detectable in the plasma of control and LPS-treated mice. In our study we found that aspirin administration *in vivo* could effectively inhibit COX-2 without increasing plasma levels of ATL. Clearly others have found aspirin to increase ATL in rodents (20, 21), which may well have utilized models where COX-2 and 5-LO were optimized. However, our study suggests that in a controlled model where low COX-2 (control mice) and increased COX-2 (LPS-treated mice) are directly compared, plasma levels of ATL do not correlate with COX-2 step changes. Aspirin treatment did significantly increase COX-2 mediated ATL in the lungs of the same mice and this correlated with COX-2 induction; however, the usefulness of lung ATL as a biomarker of COX-2 expression is questionable given that more direct measures are possible where tissue is available. Taken together, these data do not support the concept that plasma ATL can be used as a biomarker for the noninvasive assessment of vascular COX-2 expression in patients.
